# Abscopal Effect After Palliative Radiotherapy in a Patient with a Gastric Adenocarcinoma Disseminated to Retroperitoneal Space: Case Report from a Latin American Reference Center and Review of the Literature

**DOI:** 10.7759/cureus.6235

**Published:** 2019-11-26

**Authors:** Carlos E Bonilla, José Esguerra, Sara Mendoza Díaz, Angelina Álvarez, Laura Morales R

**Affiliations:** 1 Oncology, Instituto Nacional de Cancerologia, Bogota, COL; 2 Radiation Oncology, Instituto Nacional de Cancerologia, Bogota, COL; 3 Radiation Oncology, Instituto Nacional de Cancerología, Bogota, COL

**Keywords:** radiotherapy, immunotherapy, gastric carcinoma, retroperitoneal metastasis, abscopal

## Abstract

Radiation therapy is known to have a highly effective local and regional effect for cancer treatment; however, sporadic events of tumor regression in non-irradiated and irradiated fields have been observed over time, which is known as the “abscopal effect.” In this report, we describe the case of a patient with a diagnosis of unresectable advanced gastric adenocarcinoma, who developed extensive retroperitoneal lymph node involvement and did not accept management with chemotherapy. Primary radiotherapy at the local level was offered to control hemostasis, reaching an important span of complete remission of the disease.

## Introduction

Radiation therapy is one of the treatment pillars for cancer management, regardless of the stage of the disease. The main therapeutic effect of radiotherapy is at the local level, generating direct and indirect damage to the DNA of cells that have an accelerated replication such as tumor cells. In 1953, Mole described this phenomenon for the first time by documenting that radiotherapy is capable of stimulating the immune system by generating cytoreductive damage outside the radiation field [1]; this phenomenon is known as “the abscopal effect,” which has been described in melanoma, lung cancer, and renal carcinoma, among others. The term abscopal originates from the Latin root “ab,” which means far, and “scopus,” which means objective [2-3]. The phenomenon is that radiation-induced cell death triggers damage signals such as high mobility group box 1 protein (HMGB1) mobility, which leads to the activation of antigen-presenting cells (APC). Subsequently, the APCs present the tumor-derived antigens to the cytotoxic T lymphocytes (CTL) that will eventually destroy the target cell. Similarly, radiation therapy increases the expression of human leukocyte antigen (HLA) class I molecules that can also activate CTLs on the surface of tumor cells [2]. Currently, multiple studies are being carried out that combine radiotherapy with immunotherapy agents to improve the therapeutic effect and thus the oncological outcomes in patients with disease in advanced or recurrent stages. It is currently known that radiation stimulates the immune system and that it is capable of inducing immunogenic cell death; this synergy results in an effect at distant points of the disease [4]. However, to date, there are few cases reported in the literature describing this effect, especially in gastric tumors.

## Case presentation

**Figure 1:**
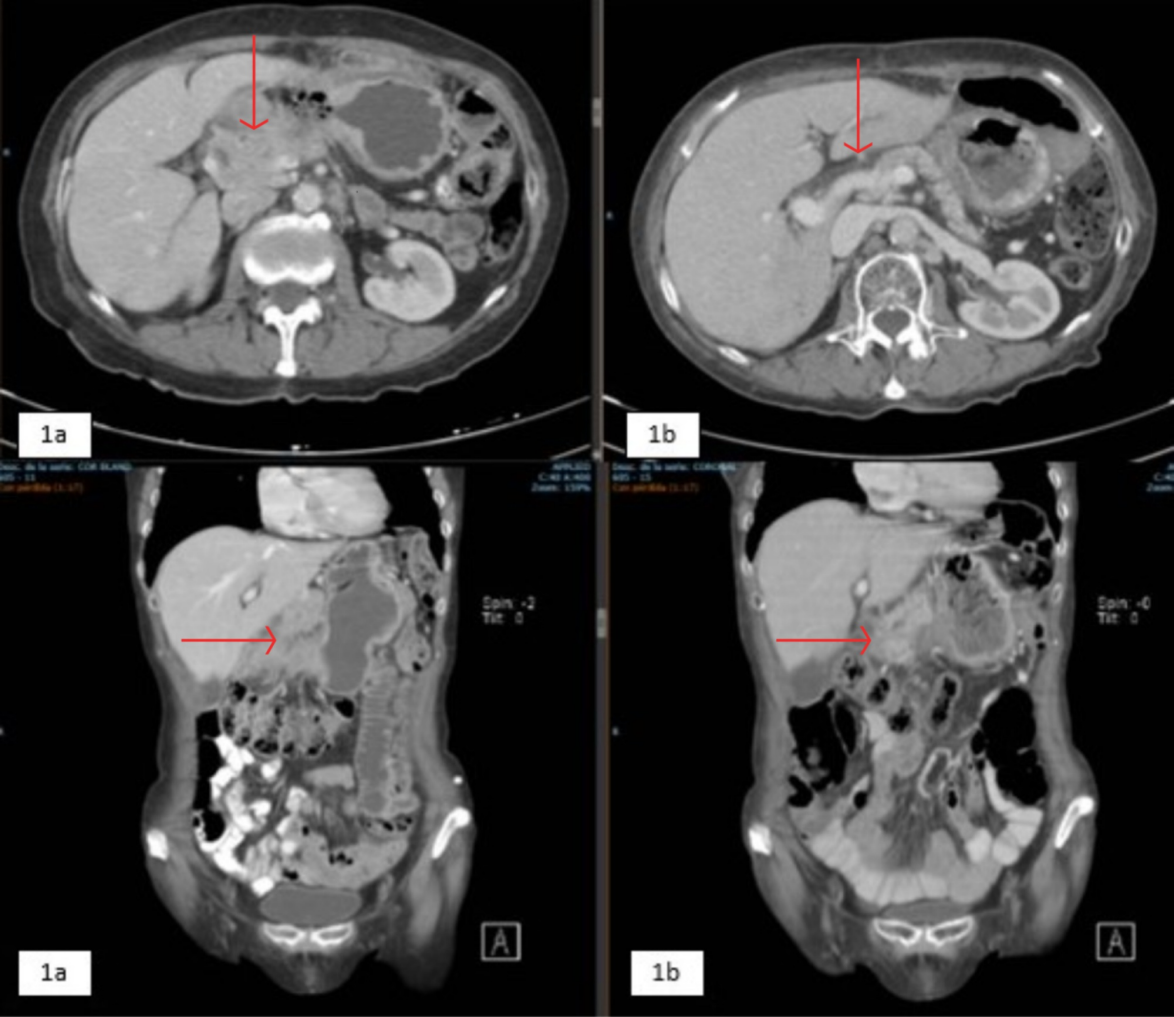
Computed tomography of the abdomen and pelvis 1a. Thickening of the neoplastic aspect in the antropyloric region of the stomach with signs of extension beyond the serosa, the involvement of the gastrocolic ligament, with suspicious retropancreatic adenomegaly, which conditions the complete occlusion of the pylorus, with gastric bypass via gastrojejunal anastomosis when compared to the previous study. 1b. Post-surgical changes due to gastric bypass and functioning intestinal anastomosis; an almost-complete resolution of the thickening of the antropyloric region walls, persisting alteration of regional fat, with no evidence of nodules. Resolution of the retroperitoneal paraaortic and gastrohepatic ligament nodal involvement.

**Figure 2:**
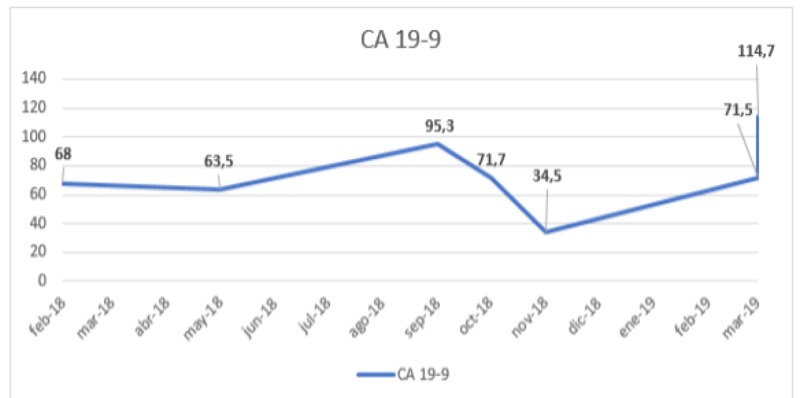
Biochemical curve: tumor marker CA-19.9 Evolutionary graphic of the tumor marker curve CA 19-9. There is a sustained response after palliative radiotherapy between the months of August 2018 and March 2019 when a significant increase in the tumor marker was documented, considering biochemical progression.

A 78-year-old woman presented with a history of high blood pressure, pulmonary thromboembolism, and papillary thyroid cancer treated with thyroidectomy and lymph node draining. At the beginning of 2016, she presented with epigastric abdominal pain, postprandial vomiting, a 6 kg loss in six months, asthenia, and adynamia. Endoscopic studies were initiated, documenting a tumor mass dependent on the gastric antrum, with a pathology report from August 2016 compatible with a well-differentiated and eroded gastric adenocarcinoma of the intestinal type, without evidence of distant metastases in CT scans. She was taken for an exploratory laparotomy, finding a large antral gastric tumor with the involvement of the pylorus and the first portion of the duodenum, without peritoneal or hepatic seeding. Due to duodenal involvement, it was considered unresectable, so palliative gastrojejunostomy was performed. Palliative chemotherapy with capecitabine and cisplatin was proposed, but the patient did not accept antineoplastic treatment, so she was treated with exclusive support care. During 2017, the disease remained stable by image, and in May 2018, it presented progression due to the increased neoplastic thickening of the stomach, the development of ganglionic metastases to the retroperitoneal space, episodes of gastrointestinal bleeding, and the elevation of tumor markers (carcinoembryonic antigen and CA 19.9). Chemotherapy was proposed again, but the patient refused treatment, so palliative radiotherapy was decided for hemostatic purposes at the stomach level. External radiotherapy is performed with the 3D conformal radiation (3DCRT) technique, prior planning, and computer simulation with the linear accelerator with 6 MV of energy in the fractionation of 3 Gy up to a total dose of 30 Gy in planning target volume-one (PTV1) (gastric mass + margin). This treatment was received between August 14 and 29, 2018, with the resolution of digestive bleeding episodes and symptomatic improvement. The November 2018 tomography scans show almost total resolution of the neoplastic thickening of the antropyloric region of the stomach, with the complete response of retroperitoneal paraaortic adenopathies and the gastrohepatic ligament (Figures 1a-1b) and the normalization of tumor markers (Table 1 and Figure 2). With these results, the patient is considered to have an abscopal effect secondary to radiotherapy treatment since the biochemical response and image response of the lesions that were inside and outside the irradiation field were achieved. The patient was free of progression until March 2019 when she showed up with multiple lymph nodes and biochemical progression and subsequently died in the month of June 2019.

**Table 1:** Biochemical curve: tumor markers ACE: angiotensin-converting enzyme

Tumor Markers	ACE (< 5.0 ng/ml)	CA 19-9 (0.0-39.0 U/ml)	CA 125 (0.0 - 35 U/ml)
23/feb/2018	2.49	68	
08/may/2018		63.5	
12/jul/2018	4.19		
07/sep/2018	6.56	95.3	
08/oct/2018	5.34	71.7	
30/nov/2018	3.43	34.5	
01/mar/2019	2.03	71.5	
27/mar/2019	2.26	114.7	
22/may/2019	6.3	5180	82.04

## Discussion

We present the case of a patient with unresectable gastric adenocarcinoma with retroperitoneal lymph node involvement who declined chemotherapy treatment. Due to the episode of digestive bleeding, palliation with radiotherapy for hemostatic purposes is performed, achieving a transient abscopal effect of eight months with the normalization of tumor markers and complete response by images, both at a gastric level and in distant metastases outside the irradiation field. Reports of cases of the abscopal effect in adenocarcinomas of the digestive tract are scarce. In the systematic review of Abuodeh et al. from 2016, 46 reports of abscopal effects were collected, of which only five corresponded to adenocarcinomas, and, of these, only one was from the digestive tract, specifically the distal esophagus [5]. Rees et al. reported this clinical case in 1982: a 49-year-old patient with distal esophageal adenocarcinoma with metastatic pulmonary involvement treated with palliative radiation therapy at 40 Gy in 20 fractions, with the subsequent disappearance of the esophageal and pulmonary lesions; this response lasted approximately 14 months [6]. In the case presented, the response lasted for approximately eight months. This difference in the response span could not be justified only regarding the difference in dose and fractionation, given that the events that occurred inside and outside the radiotherapy field, in both patients, are associated with a much more complex process of which many elements remain unknown, but it is increasingly clear that it is associated with an immune process. Radiation therapy participates in this effect by causing DNA to be released in the cytoplasm of tumor cells, leading to the synthesis of Cyclin GMP-AMP. This, together with many other mediators, allows the activation of the interferon response stimulating protein (STING), which, through diverse transcription factors and major histocompatibility complex (MHC) upregulation, results in the production of interferon type 1, which can help an antitumor immune response, which is not only limited to the irradiated site but can also track other tumor cells present in the same antigen that triggered the initial response [6-7]. Given the infrequency with which this response occurs, the number of studies that seek to enhance the chances of this happening with the use of immunotherapy has grown in recent years. Specifically, when talking about gastric cancer, the possibility of having new therapeutic tools for its management, especially in patients with locally advanced tumors, is extremely attractive, given the poor prognosis of this entity. The “ONO-4538-12, ATTRACTION-2” study phase III, published in 2017, is a clear example of the promise of immunotherapy for gastric cancer. In this study, 493 patients with gastric or gastroesophageal junction cancer, who were unresectable or recurrent, refractory, or intolerant to standard therapy, were randomized to receive nivolumab (n = 330) or placebo (n = 163). The median overall survival was 5.26 months (IC 95% 4.60 - 6.37) in the nivolumab group and 4.14 months (3.42 - 4.86) in the placebo group (HR 0.63, 95 % CI 0.51 - 0.78; p <0.0001) [8].

Currently, clinical trials are aimed at the study of these theories, which evaluate the role of radiation therapy combined with immunological therapies that could possibly achieve a therapeutic effect. A systematic review of case reports in the era of immunotherapy associated with radiotherapy evaluated the abscopal effect by identifying the dose and fractionation, sequencing, and duration of immunotherapy until the abscopal effect, calculating the biologically effective dose (BED) using the formula BED = nd [1+d / (α/ β)], where n is the number of fractions, d is the dose per fraction, and α and β are constants representing lethal and sublethal damage, respectively. It is assumed that the α/β ratio was 10 Gy, this being the typical value that generally adopts changes on the cell with rapid proliferation. The span to determine an abscopal effect is measured from the end of radiation therapy to any response in a non-irradiated site 8. Search results reported an abscopal effect after radiotherapy whether or not associated with immunotherapy in 94 cases within 52 articles, including phase I trials, retrospective series, and 48 case reports. Of the 94 patients, 47 were treated only with radiotherapy between 1969 and 2018. However, 47 cases were treated by combining immunotherapy and radiotherapy between 2012 and 2018. Some patients were excluded for not meeting the inclusion criteria: 70 patients due to concurrent chemotherapy, others for lack of sufficient information on the dose of radiotherapy, fractionation, and site, and others due to incomplete data on the characteristics of the patient and the span until an abscopal effect. Finally, 24 remaining cases were reviewed, of which 15 reports showed an abscopal effect [1]. The parameters for radiotherapy described an average total dose of 30 Gy in 5.5 fractions; the dose per average fraction was of 4.5 Gy. Schemes on fractionation were calculated in four groups:

1) Conventional fraction (1.8-2 Gy/fr); 2) Moderate hypofractionation (3-6 Gy/fr); 3) Hypofractionation (7-10 Gy) y; and 4) Ablative Dose (>12 Gy) in two different sites.

Most of the cases (23 out of 24) with an abscopal response occurred in patients who received radiotherapy in a concurrent manner or immediately after immunotherapy; one of these subsequently received treatment with Bacillus Calmette-Guerin (BCG) [9]. Finally, the conclusion was that of the 24 patients, seven demonstrated complete response, 15 demonstrated partial response, two had stable diseases, and there was a regression rate in only two cases. Sharabi et al. reported that the regression rate was 95% and the majority of patients classified with partial response had minimal disease [9]; the predominant histology was immunogenic tumors, such as melanoma, renal cell carcinoma, and lymphoma, but, in this review, a remarkable response was observed in other histological types such as gastric, esophageal, and pancreatic cancer. This suggests that the combination with these therapies may have an effective response in the poorly immunogenic for generating an abscopal effect. Different radiotherapy techniques and regimens, such as intensity-modulated radiotherapy (IMRT) or stereotactic body radiation therapy (SBRT)/stereotactic radiosurgery (SRS), could have an even greater effect on this phenomenon with an adequate selection of patients [10].

The combination of radiotherapy with colony-stimulating factors, such as granulocytes and macrophages, has also been studied as a potent stimulator of dendritic cell maturation, generating benefit from the pro-immunogenic effects associated with radiation. An analysis by Golden et al. published in The Lancet evaluated objective abscopal responses in metastatic solid tumors with promising future discussions for the creation of specific vaccines [11].

Radiation therapy is known to have a highly effective local and regional effect for cancer treatment; however, sporadic events of tumor regression in non-irradiated and irradiated fields have been observed over time, which is known as the “abscopal effect.” Evidence suggests that immunogenic cell death caused by irradiation could be another important effect of radiation therapy, in addition to DNA damage. Currently, the data found in the literature are scarce. In this new era of immunotherapy, it has been associated that this effect has an antitumor response induced by the effect of ionizing radiation, thus defining a new role for systemic radiotherapy. To date, the optimal dose, the number of fractions to achieve this effect, the joint role that checkpoint inhibitors PD-L1 and CTLA4 may have, adoptive cell therapy, or BCG vaccines cannot be clearly known. These could be related to greater power in immune activation for clinically turning into a relevant response for the patient. Recent studies evaluate the possibility that these immune responses require hypofractionation in order to elicit an effective antitumor response. These treatments with synergistic tumor effects are encouraging due to their effective potential to improve overall survival and progression-free survival; however, many challenges remain for identifying prediction biomarkers that lead to the real efficacy of this treatment and subsequent analysis of relevant and efficient clinical data that generates evidence-based guidelines [12]. It should be noted that most of the responses analyzed during the case reports described have been with immunogenic tumors, such as melanoma, renal cell carcinoma, and lymphomas, but it has also been reported in different types of tumor biologies, which suggests a potential application in other types of oncological pathologies, as well as future considerations on the frequency, dose, and durability of the treatment and its impact on oncological outcomes.

## Conclusions

The abscopal effect associated with radiotherapy in advanced cancers requires more research to determine criteria for patient choice, the optimal doses and fractionation of radiotherapy, as well as the combination with other treatments that improve the results, especially now that immunotherapy has emerged as another of the pillars of cancer treatment.
